# Germline biallelic *BRCA2* pathogenic variants and medulloblastoma: an international cohort study

**DOI:** 10.1186/s13045-024-01547-4

**Published:** 2024-04-29

**Authors:** Svenja Kastellan, Reinhard Kalb, Bia Sajjad, Lisa J. McReynolds, Neelam Giri, David Samuel, Till Milde, Miriam Elbracht, Susanne Holzhauer, Marena R. Niewisch, Christian P. Kratz

**Affiliations:** 1 Pediatric Hematology and Oncology, Hannover Medical School, Hannover, Germany; 2 Department of Human Genetics, University of Würzburg, Biocenter, Würzburg, Germany; 3 Clinical Genetics Branch Division of Cancer Epidemiology and Genetics, National Cancer Institute, Bethesda, MD USA; 4 Department of Hematology-Oncology, Valley Children’s Hospital, Madera, CA USA; 5 Hopp Children’s Cancer Center Heidelberg (KiTZ), Heidelberg, Germany; 6 Clinical Cooperation Unit Pediatric Oncology, German Cancer Research Center (DKFZ), German Consortium for Translational Cancer Research (DKTK), Heidelberg, Germany; 7 Department of Pediatric Hematology and Oncology, Heidelberg University Hospital, Heidelberg, Germany; 8 National Center for Tumor Diseases (NCT), Heidelberg, Germany; 9 Institute for Human Genetics and Genomic Medicine, Medical Faculty, RWTH Aachen University, Aachen, Germany; 10 Department of Pediatric Hematology and Oncology, Charité University Medicine, Berlin, Germany

**Keywords:** *BRCA2*, Fanconi anemia, Medulloblastoma

## Abstract

Constitutional heterozygous pathogenic variants in genes coding for some components of the Fanconi anemia-BRCA signaling pathway, which repairs DNA interstrand crosslinks, represent risk factors for common cancers, including breast, ovarian, pancreatic and prostate cancer. A high cancer risk is also a main clinical feature in patients with Fanconi anemia (FA), a rare condition characterized by bone marrow failure, endocrine and physical abnormalities. The mainly recessive condition is caused by germline pathogenic variants in one of 21 FA-BRCA pathway genes. Among patients with FA, the highest cancer risks are observed in patients with biallelic pathogenic variants in *BRCA2* or *PALB2*. These patients develop a range of embryonal tumors and leukemia during the first decade of life, however, little is known about specific clinical, genetic and pathologic features or toxicities. Here, we present genetic, clinical, pathological and treatment characteristics observed in an international cohort of eight patients with FA due to biallelic *BRCA2* pathogenic variants and medulloblastoma (MB), an embryonal tumor of the cerebellum. Median age at MB diagnosis was 32.5 months (range 7–58 months). All patients with available data had sonic hedgehog-MB. Six patients received chemotherapy and one patient also received proton radiation treatment. No life-threatening toxicities were documented. Prognosis was poor and all patients died shortly after MB diagnosis (median survival time 4.5 months, range 0–21 months) due to MB or other neoplasms. In conclusion, MB in patients with biallelic *BRCA2* pathogenic variants is a lethal disease. Future experimental treatments are necessary to help these patients.

To the editor,

The Fanconi anemia-BRCA pathway is a DNA-repair pathway that plays a role in the development of human cancer. Heterozygous germline pathogenic variants (PVs) in specific genes of this pathway are associated with an increased risk for several cancer entities, including breast, ovarian, prostate, and pancreatic cancer [1]. The same pathway is mutated in patients with Fanconi anemia (FA), a rare genetic condition defined by cellular hypersensitivity to DNA cross-linking agents. FA is caused by germline PVs in one of 21 genes (complementation groups A, B, C, D1, D2, E, F, G, I, J, L, N, O, P, Q, R, S, T, U, V, and W; MIM: 227650, 300514, 227645, 605724, 227646, 600901, 603467, 614082, 609053, 609054, 614083, 610832, 613390, 613951, 615272, 617244, 617883, 616435, 617247, 617243, 617784) [2]. With the exception of FA subgroups FA-B (X-linked) and FA-R (autosomal dominant), FA is a recessive condition caused by biallelic PVs disrupting the FA-BRCA pathway [2]. Patients with FA present with progressive bone marrow failure, physical and endocrine anomalies and an increased risk of cancer [2, 3]. Bone marrow failure develops during childhood, and approximately seven percent of patients progress to myeloid neoplasms by age 18 years [2]. Patients with FA have a high risk of head and neck, esophageal or anogenital squamous cell carcinomas affecting the majority of patients during adulthood [3].

Individuals with biallelic mutations in *BRCA2/FANCD1* (complementation group FA-D1) are rare and the phenotype differs from the phenotype associated with more common complementation groups [4]. FA-D1 patients, in particular, exhibit a high childhood cancer risk (> 300 fold increased risk), with cumulative cancer incidences greater than 70% until age 18 years [2, 4]. Embryonal tumors such as medulloblastoma (MB) and nephroblastoma (Wilms tumor) as well as primarily myeloid neoplasms are typical cancer types occurring in these patients [2, 4–8], which might be explained by additional function of BRCA2 in DNA fork protection and homologous recombinational repair. Little is known about the clinical characteristics of FA-D1 patients with specific cancer types. Here we present an international cohort of eight FA-D1 patients with medulloblastoma (MB) to characterize clinical, pathologic, genetic, treatment and outcome data.

Patients were enrolled in the German Cancer Predisposition Syndrome Registry (DRKS00017382), the Pediatric Precision Oncology INFORM Registry (DRKS00007623), or the National Cancer Institute Inherited Bone Marrow Failure Syndrome Cohort Study, Bethesda, MD, USA (NCT00027274). Two additional patients were diagnosed at the Valley Children’s Hospital, Madera, CA, USA. Defined clinical data points and specifics of the underlying PVs were collected and analyzed.

Clinical and genetic characteristics of FA-D1 patients with MB are summarized in Table 1. The location of PVs within the *BRCA2* gene and the clinical courses of the eight patients are visualized in Fig. 1. Median age at MB diagnosis was 32.5 months (range 7–58 months). The FA diagnosis was known before the MB diagnosis in three patients. All six MBs with available data were classified as sonic hedgehog (SHH)-MB. This finding is in agreement with previous reports [8, 9] and with data from genetically engineered models of SHH-MB showing that genomic instability promotes transformation of proliferating cerebellar granule neurons [10]. Six patients received chemotherapy with a documented dose reduction in two patients. We assume that platinum agents were avoided because crosslinking agents are known to be poorly tolerated by FA patients. Patient no. 2 received proton radiation treatment of the tumor region in addition to chemotherapy. This patient had the longest survival after MB diagnosis. No life-threatening treatment-related toxicities were documented. Prognosis was poor and all patients died during the follow-up period (median 4.5 months, range 0–21 months) because of MB related complications or additional malignancies.

**Table 1:** Molecular and clinical characteristics of patients with bi-allelic pathogenic variants in *BRAC2* and medulloblastoma

Pat. No	Sex	Age at diagnosis (months) MB/FA	*BRCA2* germline gDNA/AA change^a^	MB subtype	Stage^b^ at initial diagnosis	Residual tumor^c^	Chemotherapy	Radiation	Intention of therapy	Documented toxicities	Dose modifi-cation
1 FA160	M	29/30	1. c.1238del/ p.(Leu413Hisfs*17) 2. 2. c.−40 + 2 T > C/p.?	DMB, SHH L1: *TP53* wt, MYCN− L2: *TP3* mut, MYCN +	M0	No	VCR, VBL, HD-MTX, MTX i.th	No	Curative	Myelosuppression^d^, DILI^e^, oral mucositis^f^	No
2 FA161	M	54/55	c.9672dup/p.(Tyr3225Ilefs*30) (hom)	DMB, SHH, TP53 wt MYC/MYCN NA	M0	No	VCR, VBL, Cy, HD-MTX, MTX i.th., VA p.o	Proton therapy (54 Gy)	Curative	Myelosuppression^d^, DILI^e^	Yes
3	F	7/7	c.1773_1776del/p.(Ile591Metfs*22) (hom)	DMB, SHH, MYC/MYCN−	M1	Yes	VCR, MTX	No	Palliative	Diaper dermatitis	Yes
4	F	29/2	1. c.657_658del/p.(Val220Ilefs*4) 2. c.3264dup/p.(Gln1089Serfs*10)	DMB with focal anaplasia, SHH, MYC/MYCN−	M0	NA	HD-MTX, Tem, Iso, Bev	No	Curative	No	No
5	M	33/33	1. c.658_659del/p.(Val220Ilefs*4) 2. c.1189_1190insTTAG/p.(Gln397Leufs*3)	AMB, SHH, MYC/MYCN−	M0	No	HD-MTX, Niv	No	Palliative	No	No
6 UPN 55–1	M	58/58	1. c.5946del/p.(Ser1982Argfs*22) 2. C.9207 T > A/p.(Cys3069*)	NA	NA	–	No	No	NA	–	–
7 UPN 139–1	F	44/22	1. c.9196C > T/p.(Gln3066*) 2. C.5946del/p.(Ser1982Argfs*22)	NA	NA	No	No	No	NA	–	–
8 UPN 365–1	M	32/2	1. c.6641dup/p.(Tyr2215Leufs*10) 2. c.7007G > C/p.? (splicing)	SHH, MYC/MYCN−	NA	NA	Cy, VP16	NA	NA	Proteinuria, diarrhea	NA

–, no information because the patient did not receive any therapy; AMB, anaplastic medulloblastoma; Bev, bevacizumab; Cy, cyclophosphamide; DILI, drug-induced liver injury, DMB, desmoplastic medulloblastoma; f, female; FA, Fanconi anemia; hom, homozygous; HD, high dose; Iso, isotretinoin; i.th., intrathecal; L, lesion; m, male; MB, medulloblastoma; MTX, methotrexate; mut, mutated; MYC/MYCN −/+, not amplified/amplified; NA, not available; Niv, nivolumab; Pat. No, patient number; p.o., per oral; SHH, sonic hedgehog-activated; TEM, temsirolimus; VA, valproic acid; VBL, vinblastine; VCR, vincristine; VP16, etoposide; wt, wild type

^a^Based on NM_000059.4; ^b^Refers to Chang CH, Housepian EM, Herbert C. An operative staging system and a megavoltage radiotherapeutic technic for cerebellar medulloblastomas. *Radiology 93*. 1351–9, 1969; ^c^More than 1.5 cm^2^ after surgery; ^d^According to Common Terminology Criteria for Adverse Events Version 5.0 (2017): Platelets, hemoglobin and neutrophil count at least grade 3; ^e^Drug-induced liver injury (DILI) according to Hy’s law. Reuben A. Hy’s law. *Hepatology*. 2004;39(2):574–578. https://doi.org/10.1002/hep.20081; ^f^At least WHO grade 3. Palmer MK. WHO Handbook for Reporting Results of Cancer Treatment. *Br J Cancer*. 1982;45(3):484–485

**Fig. 1:**
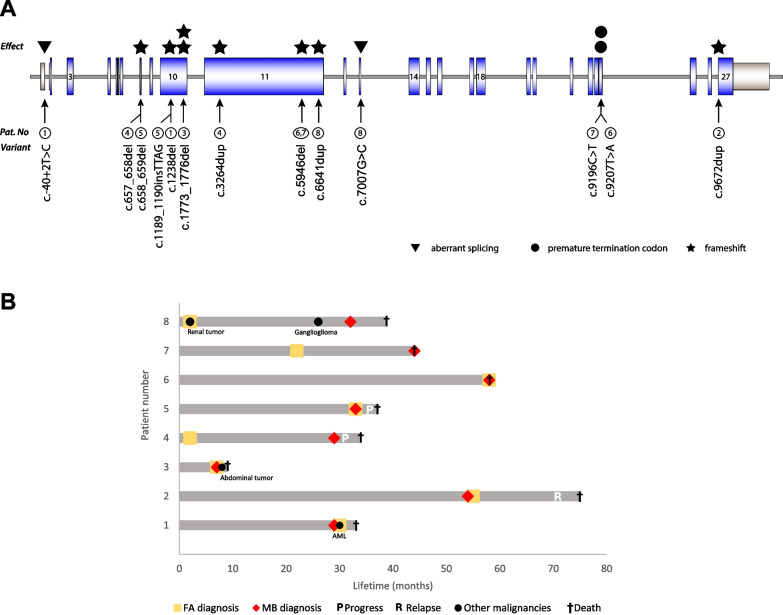
**A** Position of the germline pathogenic variants in the *BRCA2 *gene that occurred in patients with medulloblastoma. The type of variant is indicated by symbols above the representation of the *BRCA2* gene. The patient numbers are marked. **B** Swimmer plot showing the clinical course of each patient with FA-D1 and medulloblastoma. Each bar represents one patient from birth until death. Timepoints of Fanconi anemia diagnosis, other malignancies, medulloblastoma diagnosis, relapse, progress, and death are depicted

We show that MB patients with biallelic *BRCA2* pathogenic variants have an extremely poor prognosis. Limitations of this study are the small cohort size and the retrospective nature of the data collection. We recommend the development of an international consensus protocol to treat FA-D1 patients with MB, preferentially within an international clinical trial, however, due to the rarity of the condition it may be more feasible to write an international guideline developed by an international focus group. The data presented here and a previous report [11] suggest that this protocol may include radiation therapy. The use of targeted therapeutics would be ideal due to the underlying DNA repair defect, which can lead to severe chemotherapy associated toxicities. Response to olaparib has been described in a FA-A patient with metastatic esophageal squamous cell carcinoma [12], however, toxicity and efficacy of this drug in the context of FA-D1 is unknown. We recommend enrollment of FA-D1 patients in research activities, e.g. tumor signatures and patient derived xenograft models to search for novel treatments. A retrospective germline sequencing study identified four cases of FA-D1-related MB among 1022 cases of MB [9]. In five of the eight patients presented here the diagnosis of FA was unknown prior to the diagnosis of MB. The clinical consequences of such germline findings emphasize the importance of offering genetic counseling and testing to children and families with MB.

## Data Availability

All data generated or analyzed during this study are included in this published article.
